# Prognostic significance of bcl-2 expression in stage III breast cancer patients who had received doxorubicin and cyclophosphamide followed by paclitaxel as adjuvant chemotherapy

**DOI:** 10.1186/1471-2407-7-63

**Published:** 2007-04-12

**Authors:** Kyung-Hun Lee, Seock-Ah Im, Do-Youn Oh, Se-Hoon Lee, Eui Kyu Chie, Wonshik Han, Dong-Wan Kim, Tae-You Kim, In Ae Park, Dong-Young Noh, Dae Seog Heo, Sung Whan Ha, Yung-Jue Bang

**Affiliations:** 1 Department of Internal Medicine, Seoul National University College of Medicine, Seoul, Korea; 2 Department of Radiation Oncology, Seoul National University College of Medicine, Seoul, Korea; 3 Department of General Surgery, Seoul National University College of Medicine, Seoul, Korea; 4 Department of Pathology, Seoul National University College of Medicine, Seoul, Korea; 5 Cancer Research Institute, Seoul National University College of Medicine, Seoul, Korea

## Abstract

**Background:**

Bcl-2 is positively regulated by hormonal receptor pathways in breast cancer. A study was conducted to assess the prognostic significances of clinico-pathologic variables and of ER, PR, p53, c-erbB2, bcl-2, or Ki-67 as markers of relapse in breast cancer patients who had received the identical adjuvant therapy at a single institution.

**Methods:**

A cohort of 151 curatively resected stage III breast cancer patients (M:F = 3:148, median age 46 years) who had 4 or more positive lymph nodes and received doxorubicin and cyclophosphamide followed by paclitaxel (AC/T) as adjuvant chemotherapy was analyzed for clinico-pathologic characteristics including disease-free survival (DFS) and overall survival (OS). Patients with positive ER and/or PR expression received 5 years of tamoxifen following AC/T. The protein expressions of biomarkers were assessed immunohistochemically.

**Results:**

The median follow-up duration was 36 months, and 37 patients (24.5%) experienced a recurrence. Univariate analyses indicated that the tumor size (*P *= 0.038) and the number of involved lymph nodes (*P *< 0.001) significantly affected the recurrences. However, the type of surgery, the histology, histologic grade, the presence of endolymphatic emboli, and a close resection margin did not. Moreover, ER positivity (*P *= 0.013), bcl-2 positivity (*P *= 0.002) and low p53 expression (*P *= 0.032) were found to be significantly associated with a prolonged DFS. Furthermore, multivariate analysis identified 10 or more involved lymph nodes (HR 7.366; *P *< 0.001), negative bcl-2 expression (HR 2.895; *P *= 0.030), and c-erbB2 over-expression (HR 3.535; *P *= 0.001) as independent indicators of poorer DFS. In addition, bcl-2 expression was found to be significantly correlated with the expressions of ER and PR, and inversely correlated with the expressions of p53, c-erbB2 and Ki-67. Patients with bcl-2 expression had a significantly longer DFS than those without, even in the ER (+) subgroup. Moreover, OS was significantly affected by ER, bcl-2 and c-erbB2.

**Conclusion:**

Bcl-2 is an independent prognostic factor of DFS in curatively resected stage III breast cancer patients and appears to be a useful prognostic factor in combination with c-erbB2 and the number of involved lymph nodes.

## Background

Many therapeutic modalities have become available for the treatment of breast cancer. As a consequence, interest has grown for the delineation of prognostic markers identifying subsets of patients more likely to benefit from adjuvant systemic therapies and for the development of predictive markers for response to diverse treatments. Adjuvant systemic chemotherapy reduces the risk of recurrence and death in breast cancer. Taxane-containing adjuvant regimens have been reported to be more effective than anthracycline-based regimens for curatively resected node-positive breast cancer [1,2]. Doxorubicin/cyclophosphamide followed by paclitaxel (AC/T) is generally accepted as a standard for patients with node-positive breast cancer [3,4].

Analyses of the usefulness of clinico-pathologic variables as prognostic and predictive factors are clinically important, but invariably imperfect. Hormonal receptor and C-erbB2 expressions aid the selection of therapies, such as, tamoxifen and trastuzumab, and are also used to select adjuvant chemotherapies [5,6]. However, more accurate and convenient markers are needed to identify patient subgroups requiring individualized adjuvant chemotherapy.

Most anticancer agents, independently of their mechanisms of action, kill cancer cells by inducing apoptosis in response to a drug-induced damage. Alterations in the regulatory mechanisms of apoptosis are, therefore, responsible not only for the progression of breast cancer, but for different response to treatment as well [7].

Bcl-2 is a cytoplasmic protein belonging to the bcl-2 family, is expressed in normal glandular epithelium, but it is overexpressed in 25%–50% of breast cancers [8]. The bcl-2 gene was initially identified in human B-cell lymphoma because of its activity as an inhibitor of apoptosis in cancer cells. Other members of the bcl-2 family, such as bax, also promote apoptosis [9]. As a consequence, the expression of bcl-2 in cancer cells is thought to inhibit apoptosis and therefore relate to a worse outcome. However, the expression of bcl-2 in breast cancer has been found to be associated with favorable prognostic factors such as smaller tumor size, ER positivity, and low nuclear grade. Bcl-2 also predicts a more favorable outcome in metastatic disease as well as in early breast cancer patients who received heterogeneous adjuvant chemo- and hormonal therapies [10-13]. One possible explanation is that the acquisition of bcl-2 expression creates a restrictive environment for the expansion of genetically unstable and potentially malignant p53-deficient cells, causing a delay in tumor progression and explaining the different prognostic value of bcl-2 and p53 [14]. In addition, bcl-2 is known to be up-regulated by estrogen and to be down-regulated by p53 [15,16]. However, reports are contradictory concerning whether bcl-2 is an independent predictive marker for responses to primary chemotherapy [17].

In the present study, immunohistochemical staining was used to determine the prognostic significance of ER, PR, p53, c-erbB2, bcl-2, and Ki-67 expression in a homogenous patient cohort who received AC/T as adjuvant therapy for stage III breast cancer at a single institution.

## Methods

### Patients and treatment

Patients meeting the following characteristics were chosen from electronically archived medical records: (1) Curative resection of breast cancer at Seoul National University Hospital, Korea between 1999 and 2004; (2) Pathologically determined involvement of 4 or more lymph nodes; (3) The administration of AC/T as an adjuvant chemotherapy; (4) No trastuzumab adjuvant therapy; and (5) Sufficient tissue samples available for immunohistochemical analysis.

Adjuvant chemotherapy consisted of 60 mg/m^2 ^doxorubicin and 600 mg/m^2 ^cyclophosphamide every 3 weeks for 4 cycles followed by 175 mg/m^2 ^paclitaxel every 3 weeks for 4 cycles [3]. Adjuvant radiotherapy or hormonal therapy was performed as appropriate.

One hundred and fifty one patients, including 3 male patients, met these inclusion criteria and were included in this study. Patients with stage IIIB disease (T4 by AJCC staging) were not included in this study, because these patients were treated with a neoadjuvant chemotherapy protocol. Thus, all 151 patients were at stage IIIA or IIIC disease. Surgical treatment was radical modified mastectomy, without removal of the pectoralis muscles, in 120 cases (79.5%) and breast conserving surgery including quadrantectomy in 31 cases (20.5%). A level II axillary dissection was performed in all patients and the mean number of lymph nodes removed was 22.9 (range 7–54). Of the 151 patients, 89 (58.9%) were ER and/or PR positive, and 87 (97.8%) of these patients received 5 years of adjuvant tamoxifen. Thirty-one patients received breast-conserving surgery and all, except one lost to follow-up, received adjuvant radiotherapy. Of the 120 patients who underwent modified radical mastectomy, 108 received adjuvant radiotherapy. Patient characteristics are listed in Table 1.

### Pathologic Examination and Immunohistochemistry

Primary tumor specimens were paraffin-embedded after surgery. Standard histopathological examination included the type of cancer, the pathological tumor stage assessed according to the criteria established by the 6th edition of AJCC cancer staging manual [18], the grade of the tumor according to the Scarff-Bloom-Richardson (SBR) classification modified by Elston and Ellis [19], and the presence of endolymphatic emboli.

The routinely formalin-fixed, paraffin-embedded tissue blocks were sectioned at 4- μm thickness and then processed for immunohistochemistry. Tissue sections were deparaffinized in xylene, rehydrated with graded ethanol, and immersed in Tris-buffered saline. After an antigen-retrieval process, representative sections were immunostained and more than 10 randomly chosen high power fields were examined under an optical microscope.

The companies that supplied the primary antibodies and the dilution factors used were; ER (Dako Corporation, Carpinteria, CA; 1:50), PR (Dako Corporation; 1:50), p53 (Dako Corporation; 1:1200), c-erbB2 (Novocastra Laboratories Ltd., New Castle-Upon-Tyne, U.K.; 1:200), bcl-2 (Dako Corporation; 1:50), and Ki-67 (Dako Corporation; 1:800). All primary antibodies were mouse monoclonal antibodies. Biotinylated anti-mouse antibody was used as secondary antibody and streptavidin horseradish peroxidase (Zymed laboratories, San Francisco, CA) methods were used following the instructions provided by the manufacturer. Finally, the sections were counter-stained in Mayer's hematoxylin, dehydrated, cleared, and mounted for examination.

A cut-off value of 10% or more positively stained nuclei in ten high-power fields was used to define ER and PR positivity. Only cytoplasmic staining was scored as positive for bcl-2, regardless of the intensity of the stained cells. Membranous staining for C-erbB2 was scored as: 0, faint incomplete staining in 10% or less of cells; 1, faint incomplete staining in more than 10% of cells; 2, weak to moderate complete staining in more than 10% of cells; 3, strong complete staining in more than 10% of cells. Cells stained for Ki-67 were counted and expressed as percentages, and the number of cells stained for p53 were scored semi-quantitatively, as follows; 0%, 1–25%, 26–50%, 51–75%, or > 75%.

### Statistical analysis

Statistical analyses of categorical variables were performed using Pearson's χ^2 ^test or Fisher's exact test as appropriate. Disease free survival (DFS) was calculated from the date of surgery to the first observation of disease recurrence, and overall survival (OS) from the date of surgery to the date of death or the date when the patient was last known to be alive. The median durations of DFS and OS were calculated using the Kaplan-Meier method. Comparisons between groups were made using log-rank tests. Multivariate analysis was carried out using the Cox proportional hazards model. A significance level of 0.20 was used for covariate entry. Two-sided P values of < 0.05 were considered significant. All analyses were performed using SPSS for Windows, version 12.0 (SPSS Inc, Chicago, IL).

### Ethics

The study protocol was reviewed and approved by the institutional review board of Seoul National University Hospital, and complied with the recommendations of the Declaration of Helsinki for biomedical research involving human subjects.

## Results

### Patient characteristics

Baseline patient characteristics are presented in Table 1.

The median follow-up duration for the 151 patients was 36 months (range 8–78). Thirty-seven (24.5%) patients experienced breast cancer recurrence, and 34 of these had distant metastases. Frequent sites of distant metastases were bone in 17 patients, lung in 11, liver in 7, and brain in 2.

### Expressions of markers and their inter-associations

Immunohistochemical results were obtained for 151 patients. ER was positive in 83 (55.0%), PR in 59 (39.1%), and bcl-2 in 92 (60.9%). P53 was expressed in more than 25% of the tumor cells in 46 patients (30.5%); C-erbB2 was 3+ in 30 patients (19.9%); and Ki-67 was 5% or more in 56 patients (37.1%). Details are shown in Table 2.

The relationships between bcl-2 and the other markers were evaluated. Ninety two % (76 of 83) of the tumors showing ER expression co – expressed bcl-2, and bcl-2 expression was found to be correlated with PR expression. However, p53 (> 25%), c-erbB2 (3+), and Ki-67 (= 5%) expression were found to be inversely correlated with bcl-2. In addition, bcl-2 (-) tumors were significantly correlated with the histologic grade III. Details are shown in Table 3.

### Univariate analysis: clinicopathological factors and molecular markers

Tumor size (3 yr DFS, 78.6% for < 5 cm vs. 42.3% for = 5 cm, *P *= 0.038) and the number of involved lymph nodes (3 yr DFS, 89.8% for 4–9 nodes vs. 52.7% for = 10 nodes, *P *< 0.001) significantly affected recurrence, but the type of surgery, the histology, histologic grade, the presence of endolymphatic emboli, and a close resection margin < 2 mm did not.

Of the molecular markers, ER (3 yr DFS, 77.2% for ER (+) vs. 64.9% for ER (-), *P *= 0.013), bcl-2 (3 yr DFS, 77.6% for bcl-2 (+) vs. 62.0% for bcl-2 (-), *P *= 0.002) and p53 (3 yr DFS, 75.5% for p53 = 25% vs. 62.1% for p53 > 25%, *P *= 0.032) were found to be significantly related to DFS, but PR, c-erbB2, and Ki-67 were not.

OS was found to be significantly related to the number of involved lymph nodes (3 yr OS, 100% for 4–9 nodes vs. 83.7% for = 10 nodes, *P *< 0.001) and histologic grade (3 yr OS, 98.4% for histologic grade I, II vs. 84.3% for III, *P *= 0.043).

**Figure 1:**
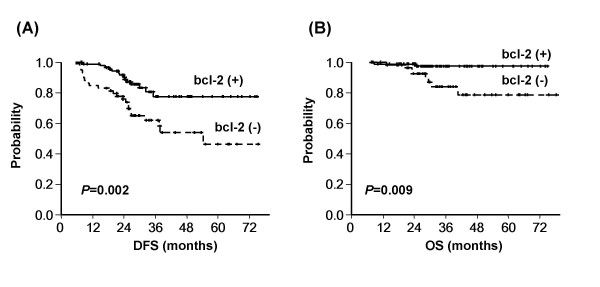
Kaplan-Meier plots of (A) disease-free and (B) overall survival according to bcl-2 expression. The expression of bcl-2 was found to be significantly and positively associated with disease-free and overall survivals.

ER (3 yr OS, 100% for ER (+) vs. 83.4% for ER (-), *P *= 0.006), bcl-2 (3 yr OS, 97.4% for bcl-2 (+) vs. 83.9% for bcl-2 (-), *P *= 0.009) and c-erbB2 (3 yr OS, 79.6% for c-erbB2 (3+) vs. 96.3% for c-erbB2 (-) to (2+), *P *< 0.001) were significantly correlated with OS (Table 4, Figure 1).

### Multivariate analysis for DFS

Multivariate analysis was performed for DFS using tumor size, number of lymph nodes, ER, PR, bcl-2, p53, c-erbB2, and Ki-67 as covariates. Ten or more lymph nodes (HR 7.366; p < 0.001), negative bcl-2 expression (HR 2.895; p = 0.033), and c-erbB2 over-expression (HR 3.535; p = 0.002) were identified as independent indicators of a shorter DFS.

### DFS according to bcl-2 status in ER (+) and ER (-) subgroups

**Figure 2:**
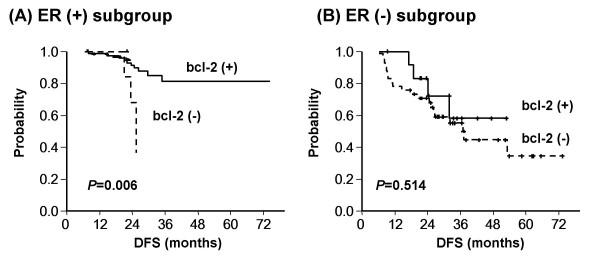
Disease-free survival according to bcl-2 status in (A) ER-positive and (B) ER-negative pateints. Bcl-2 (+) patients showed better disease-free survival even in those with an ER (+) status, suggesting that bcl-2 has a prognostic role that is independent of ER expression.

Because bcl-2 was found to be strongly correlated with ER expression, ER (+) and ER (-) subgroups were analyzed separately for DFS according to bcl-2 status. In 83 patients with ER (+) tumors, 7 patients had tumors not expressing bcl-2 and these patients had a significantly shorter DFS. However, in the ER (-) subgroup no significant differences were found on DFS according to bcl-2 status (Figure 2).

## Discussion

The identification of prognostic and predictive markers is clinically important, because breast cancer is a group of heterogenous diseases with various biological and clinical characteristics. ER and PR, as determined by IHC, have been used as predictive markers for hormonal therapy and prognostic factors. C-erbB2 status, as determined by IHC or FISH, indicates poorer survival. Possible benefits may be derived by therapeutically targeting these molecules. Recently, gene expression microarray studies have shown a strong prognostic power [20-22], but immunohistochemistry remains a convenient and powerful means of prognostication in a clinical setting as it is less expensive and easier to perform.

The present study highlights the importance of bcl-2 in breast cancer in a homogenous patient cohort. This role as an independent prognostic factor of DFS is in addition to the known prognostic factors c-erbB2 and the number of involved lymph nodes. This observation is in accordance with previous reports [10-12], and further validates the role of bcl-2 as an independent prognostic marker. However, the present study is the first to evaluate bcl-2 in high-risk patients with stage III breast cancer treated with a homogenous adjuvant regimen, i.e., AC/T. Tsutsui et al [13] recently reported that decreased bcl-2 expression is associated with a poorer disease free survival by an univariate analysis but loses its statistical significances in a multivariate analysis if proliferation activity as represented by MIB-1 counts was used as a covariate. The prognostic importance of bcl-2 in the present study is also in agreement with that of Tsutsui et al [13], but is further validated, by the multivariate analysis. Bcl-2 is a prognostic marker independent of the proliferation marker, Ki-67, possibly because of the homogeneity of the patients with 4 or more positive lymph nodes (Table 5). In addition, bcl-2 was identified in another study as a prognostic marker independent of the Nottingham prognostic index in 930 cases of mainly node-negative breast cancer with a longer follow-up period [12]. The present study reports similar results in more advanced cases with 4 or more positive lymph nodes with a shorter follow-up period.

Because bcl-2 blocks apoptosis *in vitro*, and thus contributes to malignant cell accumulation, its over-expression is expected to be associated with more aggressive tumor biology. Indeed, genetic alteration of the bcl-2 gene located on chromosome 18 is considered to be a key process in the pathogenesis and chemoresistance of human tumors, such as follicular lymphoma [23]. However, in breast cancer specimens, bcl-2 expression is associated with markers of better differentiation, like lower grade lesions, ER positivity, and a low proliferation status [24]. These aspects of bcl-2 are reproduced in the present study. In the present study, bcl-2 was identified as an independent marker of DFS by multivariate analysis when other associated factors were used as covariates. Tumors expressing ER are considered more indolent, as exemplified by 'luminal tumors' in gene expression microarray studies [20-22]. In the present study, bcl-2 was found to be a stronger predictor of DFS than ER expression, as shown in Table 4, and ER (+), bcl-2 (-) patients were observed to have a shorter DFS than ER (+), bcl-2 (+) patients. These results suggest the important role of bcl-2 expression independently of hormonal receptor markers.

However, whether bcl-2 is a predictive marker of benefit from the specific adjuvant chemotherapy AC/T, which contains paclitaxel, is not clear. This was difficult to determine in our cohort which included patients treated with a single regimen containing paclitaxel but not controls. Bcl-2 was not found to have any predictive value in terms of response to a single cycle of perioperative 5-FU/doxorubicin/cyclophosphamide chemotherapy in a previous study of 423 patients [8]. A small number of preclinical reports are available regarding taxane chemosensitivity and bcl-2 protein expression. Bcl-2 expression was found to be related to a response to paclitaxel in human non-small cell lung cancer tumors heterotransplanted into nude mice [25]. Positive bcl-2 expression was found to be significantly associated with enhanced docetaxel sensitivity *in vitro *in non-small cell lung cancer [26]. Moreover, bcl-2 down-regulation has been associated with paclitaxel resistance [27]. These preclinical findings suggest the possibility that bcl-2 is a predictive marker of response to paclitaxel, and this possibility requires further evaluation.

In the present study, C-erbB2, also called HER2, as is bcl-2 was found to be an independent predictor of a shorter DFS. C-erbB2 amplification is known to be associated with clinical responsiveness to anthracycline-containing chemotherapy [28,29]. Furthermore, the CALGB 9344 trial, which demonstrated improved outcomes for AC/T, showed that the benefit of adding paclitaxel to AC is greater for C-erbB2 (+) tumors, whereas paclitaxel showed no apparent benefit in the ER (+), c-erbB2 (-) group [3,30], which suggests the efficacy of paclitaxel against c-erbB2 (+) tumors. In the present study, c-erbB2 over-expression indicated a poorer outcome, despite the use of two active chemotherapeutic agents, doxorubicin and paclitaxel. Patients treated with adjuvant trastuzumab were not included in this study, and thus the effects of this targeting agent were excluded.

## Conclusion

We conclude that bcl-2, and the known prognostic factors c-erbB2 and the number of involved lymph nodes are independent prognostic factors of DFS in curatively resected stage III breast cancer patients, and that molecular marker analysis is useful to discriminate subsets of patient with different prognoses.

## Competing interests

The author(s) declare that they have no competing interests.

## Authors' contributions

KHL carried out the collection of the data, the statistical analysis and drafted the manuscript. SAI designed the concept of this study, performed the statistical analysis with interpretation and approved the final manuscript. DYO, SHL, DWK, TYK, DSH and YJB performed the chemotherapy for patients and revised the manuscript. EKC and SWH performed radiation therapy for patients and participated in treatment coordination. WH and DYN involved in provision of study patients and treatment coordination. IAP carried out the immunoassays and pathologic examinations. All authors were involved in the research presented and approved the final manuscript.

## Pre-publication history

The pre-publication history for this paper can be accessed here:



## Figures and Tables

**Table 1:** Clinicopathologic characteristics of 151 patients

		Pt. number	%
Sex	M	3	2.0%
	F	148	98.0%
Age	median	46 years	range 28–70 years
	≤50	111	73.5%
	> 50	40	26.5%
Surgery	MRM	120	79.5%
	BCS	31	20.5%
Histology	ductal	138	91.4%
	others	13	8.6%
Stage	IIIA	79	52.3%
	IIIC	72	47.7%
Tumor size	pT ≤ 2 cm	11	7.3%
	2 < pT ≤ 5	110	72.8%
	pT > 5 cm	30	19.9%
Involved LNs	4–9	79	52.3%
	≥10	72	47.7%

**Table 2:** Molecular characteristics of the tumors

		Pt. number	%
ER	(-)	68	45.0
	(+)	83	55.0
PR	(-)	92	60.9
	(+)	59	39.1
bcl-2	(-)	59	39.1
	(+)	92	60.9
p53	0	47	31.1
	1–25%	58	38.4
	26–50%	11	7.3
	51–75%	8	5.3
	76–100%	27	17.9
c-erbB2	0	65	43.0
	+	36	23.8
	++	20	13.2
	+++	30	19.9
Ki-67	< 5%	67	44.4
	≥5%	84	55.6

**Table 3:** The association between bcl-2 and other markers

		Bcl-2 expression		*P*
		(-)	(+)	
Histology	ductal	55 (39.9)	83 (60.1)	0.521
	others	4 (30.8)	9 (69.2)	
Histologic grade	I, II	14 (21.2)	52 (78.8)	< 0.001
	III	42 (54.5)	35 (45.5)	
Endolymphatic tumor emboli	absent	2 (33.3)	4 (66.7)	1.000
	present	43 (37.1)	73 (62.9)	
ER	(-)	52 (76.5)	16 (23.5)	< 0.001
	(+)	7 (8.4)	76 (91.6)	
PR	(-)	55 (59.8)	37 (40.2)	< 0.001
	(+)	4 (6.8)	55 (93.2)	
p53	≤25%	33 (31.4)	72 (68.6)	0.004
	>25%	26 (56.5)	20 (43.5)	
c-erbB2	(-)~(2+)	42 (34.7)	79 (65.3)	0.027
	(3+)	17 (56.7)	13 (43.3)	
Ki-67	< 5%	18 (26.9)	49 (73.1)	0.006
	≥5%	41 (48.8)	43 (51.2)	

n (%) patients for each variable. *P*-values were obtained using the chi-square test.

**Table 4:** Univariate analyses of clinicopathological factors and molecular markers

		Number	3 yr DFS	*P*	3 yr OS	*P*
Tumor size	< 5 cm ≥5 cm	121 30	78.6% 42.3%	0.038	92.1% 91.1%	0.952
Number of lymph nodes	4–9 ≥10	79 72	89.8% 52.7%	< 0.001	100% 83.7%	0.001
Surgery	MRM BCS	120 31	70.0% 79.0%	0.808	90.9% 96.3%	0.953
Histology	Ductal Others	138 13	71.1% 76.9%	0.461	91.0% 100.0%	0.323
Histologic grade	I, II III	65 76	74.7% 69.5%	0.160	98.4% 84.3%	0.043
Endolymphatic tumor emboli	absent present	5 116	55.6% 71.7%	0.459	100% 91.6%	0.490
Close RM	≤2 mm > 2 mm	5 146	70.8% 80.0%	0.590	91.5% 100%	0.475
ER	(-) (+)	68 83	64.9% 77.2%	0.013	83.4% 100%	0.006
PR	(-) (+)	92 59	68.3% 76.2%	0.136	87.1% 100%	0.121
p53	≤25% >25%	105 46	75.5% 62.1%	0.032	93.8% 87.8%	0.213
bcl-2	(-) (+)	59 92	62.0% 77.6%	0.002	83.9% 97.4%	0.009
c-erbB2	(-) to (2+) (3+)	121 30	74.3% 60.6%	0.112	96.3% 79.6%	0.050
Ki-67	< 5% ≥5%	94 56	85.6% 61.8%	0.146	91.0% 92.4%	0.631

**Table 5:** Multivariate analysis for disease free survival

	HR	95% CI	*P*-value
tumor size (≥ 5 cm)	1.260	(0.602, 2.637)	0.539
number of lymph nodes (≥ 10)	7.366	(2.982, 18.197)	< 0.001
ER (-)	0.988	(0.316, 3.089)	0.984
PR (-)	0.717	(0.254, 2.025)	0.530
bcl-2 (-)	2.895	(1.090, 7.690)	0.033
p53 (> 25%)	1.065	(0.507, 2.237)	0.868
c-erbB2 (3+)	3.535	(1.616, 7.731)	0.002
Ki-67 (≥ 5%)	1.247	(0.614, 2.534)	0.541
